# The role of gut microbiota dysbiosis in the pathogenesis of hyperuricemic nephropathy

**DOI:** 10.3389/fmolb.2026.1872377

**Published:** 2026-06-24

**Authors:** Quanli Jiang, Xiaolin Zhu, Liping Yin

**Affiliations:** The Second Affiliated Hospital of Nanjing University of Chinese Medicine, Nanjing, China

**Keywords:** dysbiosis, gut microbiota, gut-kidney axis, hyperuricemic nephropathy, uric acid metabolism

## Abstract

Hyperuricemic nephropathy (HN) is a renal complication associated with sustained hyperuricemia and urate-related renal injury. Emerging evidence suggests that gut microbiota dysbiosis may participate in HN pathogenesis by influencing uric acid metabolism, intestinal urate excretion, gut barrier integrity, microbial metabolite production, and gut-kidney immune crosstalk. However, the strength of evidence varies substantially across proposed mechanisms, with many findings derived from animal models, *in vitro* experiments, CKD studies, or human studies of hyperuricemia and gout rather than HN-specific clinical cohorts. This review summarizes current clinical and experimental evidence linking gut microbiota dysbiosis with HUA, gout, CKD, and HN, critically evaluates proposed mechanistic pathways, and discusses microbiota-targeted interventions including probiotics, prebiotics, dietary strategies, fecal microbiota transplantation, and metabolite-based approaches. Particular emphasis is placed on distinguishing association from causality and identifying translational gaps that should be addressed in future HN-specific studies.

## 1 Introduction

With changes in dietary patterns and lifestyle, the global incidence of hyperuricemia-induced hyperuricemic nephropathy has increased year by year, and HN has become an important contributor to chronic kidney disease (CKD) and end-stage renal disease (ESRD) (Stack et al., 2019). Traditional studies on the pathogenesis of HN have largely focused on the renal parenchyma, proposing that urate crystal deposition in the renal interstitium activates the NLRP3 inflammasome, promotes the release of pro-inflammatory cytokines such as IL-1β and IL-6, induces apoptosis of renal tubular epithelial cells, and ultimately may participate in renal injury (Mei et al., 2022). However, clinical observations show that renal injury continues to progress in some patients even after serum uric acid has been controlled, suggesting the presence of key regulatory pathways beyond the kidney (Zho et al., 2024).

The gut microbiota refers to the collective microorganisms colonizing the human intestinal tract. Its cell number is approximately ten times that of human cells, and its gene content is estimated to be around 100 times that of the human genome. It plays a central role in substance metabolism, immune regulation, and barrier protection. Under physiological conditions, the gut microbiota and the kidney maintain bidirectional homeostasis through the gut-kidney axis. The kidney provides a suitable microenvironment for the gut microbiota by regulating water-salt metabolism and acid-base balance, whereas the gut microbiota protects renal function through metabolites and immune regulation (Chen et al., 2022). When the structure and function of the gut microbiota become disturbed, gut-kidney axis homeostasis is disrupted, and multiple pathways may participate in the pathogenesis of HN.

Existing studies have reported significant gut microbiota dysbiosis in patients with hyperuricemia and HN, and the degree of dysbiosis is positively correlated with markers of renal injury (Zho et al., 2024). Therefore, clarifying the role of gut microbiota dysbiosis in the pathogenesis of HN is crucial for advancing pathophysiological understanding and developing new intervention strategies.

**FIGURE 1:**
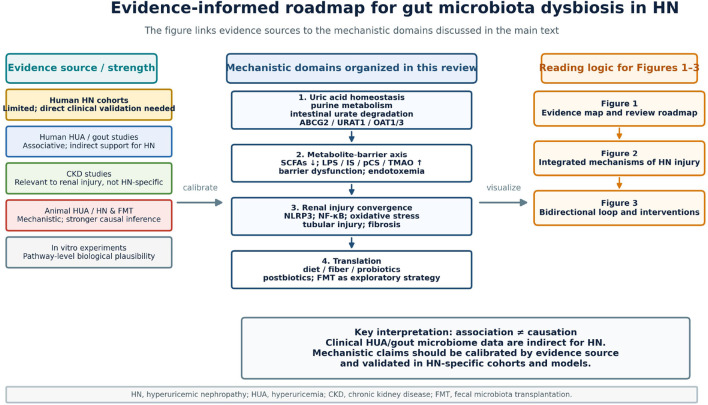
Evidence-informed graphical roadmap for gut microbiota dysbiosis in hyperuricemic nephropathy. Current evidence should be interpreted according to its source and strength. Direct human data in clinically defined HN remain limited, whereas human HUA/gout studies, CKD studies, animal HUA/HN models, fecal microbiota transplantation experiments, and *in vitro* studies provide indirect or mechanistic support. The figure links these evidence layers to three major domains used in this review: urate homeostasis, gut-derived metabolites and barrier dysfunction, and bidirectional gut-kidney feedback with intervention strategies. HN, hyperuricemic nephropathy; HUA, hyperuricemia; CKD, chronic kidney disease; FMT, fecal microbiota transplantation.

**FIGURE 2:**
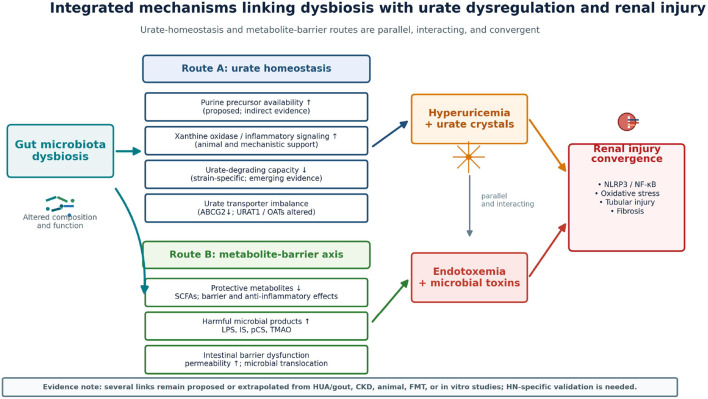
Integrated mechanisms linking gut microbiota dysbiosis with urate dysregulation and renal injury in hyperuricemic nephropathy. Dysbiosis may affect HN through two interconnected routes. The urate-homeostasis route includes altered purine substrate availability, xanthine oxidase and inflammatory signaling, reduced strain-specific urate degradation, and altered intestinal or renal urate transporter expression. The metabolite-barrier route includes decreased SCFAs, increased LPS, IS, pCS, and TMAO, intestinal barrier disruption, microbial translocation, TLR4/NF-kappaB/NLRP3 activation, oxidative stress, tubular injury, and fibrosis. Several links are extrapolated from HUA, gout, CKD, animal, FMT, or *in vitro* studies and require HN-specific validation. SCFA, short-chain fatty acid; LPS, lipopolysaccharide; IS, indoxyl sulfate; pCS, p-cresyl sulfate; TMAO, trimethylamine N-oxide; NF-kappaB, nuclear factor-kappaB; NLRP3, NOD-like receptor family pyrin domain containing 3.

**FIGURE 3:**
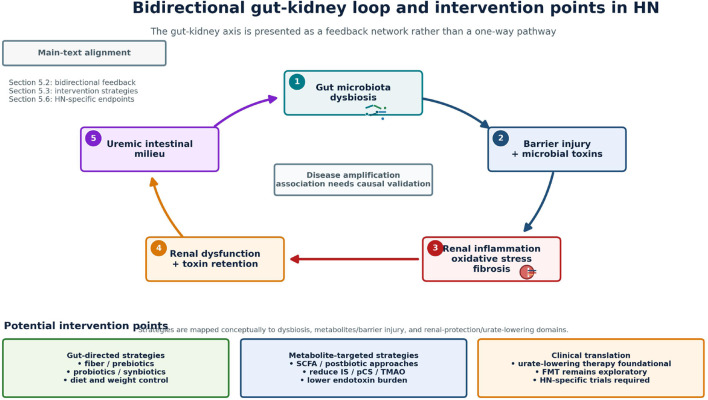
Bidirectional gut-kidney feedback loop and potential microbiota-targeted intervention points in hyperuricemic nephropathy. Gut dysbiosis may promote hyperuricemia, barrier injury, microbial toxin accumulation, inflammation, oxidative stress, and renal fibrosis; renal dysfunction may in turn reshape the intestinal environment through toxin retention, uremia-related epithelial stress, altered urea metabolism, diet or medication exposure, and systemic inflammation. Potential intervention points include conventional urate-lowering therapy, dietary modification and fiber or prebiotics, probiotics or synbiotics, postbiotic or metabolite-targeted strategies, and FMT as an experimental or future strategy. The clinical efficacy of these approaches in HN remains to be established. FMT, fecal microbiota transplantation; HN, hyperuricemic nephropathy; SCFA, short-chain fatty acid.

Consistent with this evidence-based framework, the graphical presentation in this review has been organized in a stepwise sequence: the evidence base is first mapped, then the integrated mechanistic pathways are summarized, and finally the bidirectional gut-kidney loop and potential intervention points are shown (Figures 1–3). This structure is intended to improve text-figure consistency and to prevent overlap among the three figures.

## 2 Clinical evidence and evidence gaps in human studies

Although increasing clinical studies have linked gut microbiota dysbiosis to hyperuricemia and gout, direct human evidence specifically focusing on hyperuricemic nephropathy remains limited. Most available human microbiome studies have investigated asymptomatic hyperuricemia or gout rather than HN with clearly defined renal pathological or functional outcomes. Therefore, evidence from HUA and gout populations should be interpreted as indirect clinical support for HN-related mechanisms, rather than as definitive proof of causality in HN.

Several human studies have reported altered gut microbial composition in individuals with HUA or gout. These studies have identified changes in microbial diversity, enrichment or depletion of specific bacterial taxa, and alterations in fecal metabolites or predicted microbial functions related to purine metabolism, urate handling, inflammation, and short-chain fatty acid production. However, the reported microbial signatures are not fully consistent across studies, probably because of differences in ethnicity, diet, medication exposure, disease stage, sample size, sequencing strategy, and statistical methods. Representative human microbiome studies related to hyperuricemia and gout and their relevance to HN are summarized in Table 1.

**TABLE 1:** Representative human microbiome studies related to hyperuricemia and gout and their relevance to hyperuricemic nephropathy.

Study	Population	Disease category	Study design/Method	Main microbiota-related findings	Relevance to HN	Main limitations
Shao et al. (2017)	26 male gout patients and 26 healthy controls	Gout	Fecal 16S rRNA sequencing + fecal metabolomics	Distinct fecal microbial and metabolomic profiles; enrichment of opportunistic taxa and altered metabolites related to purine metabolism and inflammation	Indirect clinical evidence linking dysbiosis with urate-related disease	Small male-only cohort; gout rather than HN; renal outcomes not central
Chu et al. (2021)	102 gout patients and 86 healthy controls; 307 fecal samples	Gout	Shotgun metagenomic sequencing	Gout metagenomes differed from controls; microbial gene profiles showed diagnostic potential	Supports functional alteration of the gut microbiome in urate-related disease	Gout-focused; kidney-specific outcomes limited; cross-sectional inference
Kim et al. (2022b)	Asymptomatic HUA, gout, and healthy controls	Asymptomatic HUA/gout	Fecal 16S rRNA sequencing	Microbial composition differed among asymptomatic HUA, gout, and controls	Suggests HUA and gout should not be treated as identical when extrapolating to HN.	Cross-sectional; not HN-specific; causality cannot be inferred
Martínez-Nava et al. (2023)	Asymptomatic HUA, gout, and healthy controls	Asymptomatic HUA/gout	16S rRNA sequencing + predicted functional analysis	Differences in SCFA-producing bacteria and predicted microbial functions were reported	Relevant to proposed SCFA, barrier, and inflammation-related mechanisms	Predicted function only; no direct metabolomics or HN endpoints
Miyajima et al. (2024)	400 Japanese adults after exclusions	HUA vs. non-HUA	Fecal 16S rRNA sequencing + machine learning + causal inference modeling	Lower Shannon diversity in HUA; Collinsella and Dorea were associated with serum uric acid	Supports an association between gut microbiota and serum uric acid in humans	Observational; few HUA participants; no direct HN diagnosis or outcome

Abbreviations: HN, hyperuricemic nephropathy; HUA, hyperuricemia; SCFA, short-chain fatty acid; SUA, serum uric acid.

These studies mainly provide indirect clinical evidence for HN because most were conducted in individuals with HUA or gout rather than patients with clinically defined HN. Therefore, their findings should be interpreted as associative rather than causal.

Importantly, most clinical studies are cross-sectional and cannot determine whether gut microbiota dysbiosis is a cause, consequence, or modifier of hyperuricemia-related renal injury. Animal models and fecal microbiota transplantation experiments provide stronger mechanistic support, but their findings require careful translation to human HN. Accordingly, in the following mechanistic sections, we distinguish evidence derived from HN-specific models, HUA or gout populations, CKD studies, animal experiments, and *in vitro* mechanistic studies whenever possible.

Figure 1 provides an evidence-informed roadmap for the remainder of this review. It separates direct HN-related evidence from indirect evidence derived from HUA/gout populations, CKD studies, animal models, fecal microbiota transplantation experiments, and *in vitro* studies, and links these evidence layers to the main mechanistic domains discussed below.

## 3 Direct regulation of uric acid metabolism by gut microbiota dysbiosis

### 3.1 Promotion of endogenous uric acid production

#### 3.1.1 Direct regulation of purine synthesis pathways by dysbiosis

Endogenous uric acid production mainly depends on two pathways: the *de novo* purine synthesis pathway, which uses small molecules such as amino acids as substrates, and the salvage synthesis pathway, which recycles free purine bases into nucleotides (Yamaoka et al., 1996). Traditionally, endogenous uric acid production has been considered to be primarily regulated by the host’s own genetic background and metabolic state (Leask et al., 2020). However, with rapid advances in metagenomics and microbiomics, increasing evidence indicates that the gut microbiota, as the host’s “second genome”, can profoundly influence purine metabolism and uric acid homeostasis through complex metabolic networks and immune-regulatory functions (Sun et al., 2025). When dietary imbalance, antibiotic overuse, intestinal infection, environmental stress, or related factors disrupt the structure and function of the gut microbiota, its normal regulatory effects on purine metabolism may fail and instead become an important driver of increased endogenous uric acid production (Sun et al., 2025; Shao et al., 2017).

The composition and metabolic activity of the gut microbiota directly affect the decomposition and absorption of nutrients in the intestine (Schoeler and Caesar, 2019). In a healthy state, beneficial bacteria such as Bifidobacterium and *Lactobacillus* predominate. The enzymes they secrete moderately degrade food residues and exfoliated intestinal cells, thereby providing essential nutrients to the host while preventing excessive accumulation of metabolic by-products (ojo et al., 2014; Maske et al., 2021). In dysbiosis, however, the abundance of harmful bacteria such as Enterobacteriaceae increases markedly, and their metabolic behavior changes. Dysbiosis may theoretically increase the availability of amino-acid substrates involved in purine biosynthesis; however, direct evidence demonstrating that this pathway increases endogenous uric acid production in human HN remains limited (Song et al., 2022).

Excessive proliferation of harmful bacteria markedly increases the activity of secreted proteases (Rondeau et al., 2024). These proteases excessively degrade structural proteins such as collagen and elastin in the intestinal mucosal layer, as well as dietary proteins, releasing large amounts of glycine, glutamine, aspartate, and other amino acids. These amino acids are key precursors in the *de novo* purine synthesis pathway: glycine provides C4, C5, and N7 of the purine ring; glutamine provides N3 and N9; and aspartate provides N1 (Baggott and Tamura, 2015; Cader et al., 2020). After intestinal absorption, these precursors enter the bloodstream and are transported to organs such as the liver, supplying abundant substrates for *de novo* purine synthesis (Houlier et al., 1991). As the major site of endogenous purine synthesis, the liver shows a markedly accelerated purine synthesis rate when substrates are sufficient, which may contribute to increased endogenous uric acid production under certain experimental or metabolic conditions (Itakura et al., 1986).

In addition, dysbiosis may impair intestinal absorption and metabolism of nutrients such as folate and vitamin B12. As cofactors involved in purine synthesis and metabolism, impaired absorption of these nutrients may indirectly disturb purine metabolic balance and further exacerbate endogenous uric acid production (Baggott and Tamura, 2015).

Beyond the *de novo* synthesis pathway, the purine salvage pathway is also an important source of endogenous uric acid. This pathway enzymatically recycles free purine bases and nucleosides, such as adenine and hypoxanthine, into nucleotides, thereby reducing purine loss (Edwards et al., 1979). Under healthy conditions, adenosine deaminase (ADA) expressed by intestinal epithelial cells degrades excess adenosine into hypoxanthine, which is further metabolized by xanthine oxidase into uric acid. This process removes excess adenosine while also providing essential nucleotides through the salvage pathway to maintain purine metabolic balance (Xu and Kellems, 2000). During dysbiosis, however, disordered microbial communities and their metabolites may inhibit ADA activity in intestinal epithelial cells (de Araújo Junqueira et al., 2011), causing intracellular adenosine accumulation and feedback activation of key enzymes in the purine salvage pathway, namely adenine phosphoribosyltransferase (APRT) and hypoxanthine-guanine phosphoribosyltransferase (HGPRT). Enhanced activity of these enzymes promotes the combination of free purine bases with 5′-phosphoribosyl-1-pyrophosphate (PRPP) to synthesize nucleotides. Some nucleotides are further degraded to produce more hypoxanthine and xanthine, which are ultimately catalyzed by xanthine oxidase to generate uric acid, thereby increasing endogenous uric acid levels (Simmonds et al., 1978; Sauer et al., 2012). Meanwhile, dysbiosis may affect the activity of phosphoribosyl pyrophosphate synthetase (PRPS), a shared key enzyme in both *de novo* and salvage purine synthesis. Abnormally increased PRPS activity can provide more PRPP for both pathways, further promoting endogenous uric acid production (Choi et al., 2020; Zhen et al., 2022). The potential involvement of ADA, APRT, HGPRT, and PRPS in dysbiosis-related purine metabolism should be interpreted as a mechanistic hypothesis. At present, direct evidence linking gut microbiota dysbiosis to these enzymatic changes in patients with HN is insufficient.

#### 3.1.2 Uric acid elevation mediated by inflammation and energy-metabolism disorders

Gut microbiota dysbiosis not only directly affects purine metabolism but also indirectly promotes endogenous uric acid production by inducing chronic low-grade inflammation (Sun et al., 2025). Under physiological conditions, the gut microbiota maintains intestinal barrier integrity through metabolites such as short-chain fatty acids (SCFAs). SCFAs promote proliferation and differentiation of intestinal epithelial cells, strengthen the structure and function of tight-junction proteins such as occludin and ZO-1, and effectively prevent harmful substances such as lipopolysaccharide (LPS) from entering the circulation (Liu S. et al., 2025; Fu et al., 2023).

In dysbiosis, the reduction of beneficial bacteria decreases SCFA production, while the expansion of harmful bacteria further disrupts barrier function (Maciel-Fiuza et al., 2023). Increased intestinal permeability allows endotoxins and other microbial-associated molecular patterns (MAMPs) to enter the bloodstream, inducing systemic chronic low-grade inflammation (de Punder and Pruimboom, 2015). Once in the circulation, endotoxins bind to Toll-like receptor 4 (TLR4) on immune cells and activate the nuclear factor-κB (NF-κB) signaling pathway, promoting the expression and release of inflammatory mediators such as IL-1β, IL-6, and TNF-α. These mediators not only intensify inflammation but also alter the activity of enzymes involved in purine metabolism (Guijarro-Muñoz et al., 2014). On the one hand, inflammatory mediators activate xanthine oxidase, accelerating the conversion of hypoxanthine and xanthine into uric acid; as a key enzyme in uric acid production, increased xanthine oxidase activity directly enhances endogenous uric acid synthesis (Wang et al., 2022). On the other hand, inflammation induces cellular injury and apoptosis, leading to the extracellular release of nuclear DNA and RNA. As major storage forms of purines, nucleic acids are degraded by nucleases into purine bases and enter purine metabolism, providing additional substrates for endogenous uric acid production (Shi, 2010).

The gut microbiota is closely associated with host energy metabolism. Its metabolites regulate host lipid synthesis, glucose metabolism, and energy expenditure through multiple signaling pathways (Amabebe et al., 2020). Dysbiosis disrupts this regulatory function and indirectly affects purine metabolism and endogenous uric acid production (Sun et al., 2025). AMP-activated protein kinase (AMPK) is a core regulator of cellular energy metabolism, and its activity is precisely controlled by gut microbial metabolites. Under physiological conditions, indole compounds produced by the gut microbiota activate hepatic AMPK, inhibit lipid-synthesis enzymes such as acetyl-CoA carboxylase (ACC), reduce lipid synthesis and accumulation, promote fatty-acid oxidation, and maintain cellular energy homeostasis (Choi et al., 2013). In dysbiosis, the increased abundance of harmful bacteria decreases indole production, reduces hepatic AMPK activity, relieves inhibition of lipid-synthesis enzymes, increases hepatic lipid synthesis, and induces metabolic disorders such as non-alcoholic fatty liver disease (Ding et al., 2024).

Reduced AMPK activity aggravates intracellular oxidative stress, damages cellular DNA and RNA, and induces apoptosis (Zhu et al., 2024; Sinha et al., 2013). Nucleic acids released from apoptotic cells are degraded into purine bases and participate in metabolism, ultimately promoting endogenous uric acid production (Kono et al., 2010).

In addition, dysbiosis may indirectly regulate purine metabolism by affecting insulin signaling. Insulin resistance, a core feature of metabolic syndrome, is closely associated with hyperuricemia (Asma Sakalli et al., 2023). Gut microbiota dysbiosis can aggravate insulin resistance by altering intestinal hormone secretion and inflammatory responses. Insulin resistance not only impairs renal uric acid excretion but may also promote endogenous uric acid production by regulating the activity of enzymes involved in purine metabolism (Zhang Y. et al., 2025).

### 3.2 Inhibition of intestinal uric acid excretion

#### 3.2.1 Reduced uric acid degradation in the intestine due to loss of beneficial bacteria

Under physiological conditions, the gut microbiota actively participates in and promotes intestinal uric acid excretion through multiple synergistic mechanisms, thereby maintaining dynamic homeostasis of host uric acid metabolism.

First, some intestinal bacterial strains harbor urate-degrading enzymes or functional pathways that may contribute to intestinal urate metabolism. These microbial processes may transform urate into more soluble downstream products and thereby support intestinal elimination. However, this effect appears to be strain-specific, and its quantitative contribution to urate lowering in human HN remains insufficiently defined (Liu et al., 2023). This process may lower intestinal uric acid concentration but also reduces reabsorption of uric acid through intestinal epithelial cells back into the circulation. Second, a healthy gut microbiota is essential for maintaining intestinal barrier integrity. It competitively inhibits the colonization of harmful bacteria and secretes antibacterial substances such as bacteriocins to suppress pathogenic growth (Fu et al., 2023). Meanwhile, SCFAs generated by microbial metabolism provide energy to intestinal epithelial cells and promote the expression of tight-junction proteins such as occludin and claudins, collectively enhancing intestinal barrier integrity, preventing LPS and other endotoxins from entering the bloodstream, and reducing the interference of systemic inflammation with uric acid excretion (Liu et al., 2024). In addition, intestinal epithelial cells express multiple uric acid transporters, including urate anion transporter 1 (URAT1), which mediates uric acid reabsorption, and ATP-binding cassette transporter G2 (ABCG2), which mediates uric acid secretion (Yano et al., 2014). Gut microbiota-derived metabolites, particularly SCFAs, have been proposed to modulate urate transporter expression and activity. However, most available evidence is derived from experimental models or non-HN conditions, and direct validation in human HN remains limited. For example, SCFAs activate G protein-coupled receptor signaling pathways, including GPR43 and GPR41, downregulating intestinal epithelial URAT1 and upregulating ABCG2, thereby promoting uric acid secretion from epithelial cells into the intestinal lumen and reducing uric acid reabsorption (Kim et al., 2011).

#### 3.2.2 Imbalance of uric acid transporters aggravates uric acid reabsorption

When the composition and function of the gut microbiota are disrupted, however, its uric-acid-excretory effect is weakened or even reversed, suppressing intestinal uric acid excretion and increasing serum uric acid. A plausible feature of dysbiosis is the loss of bacteria with urate-degrading potential or reduced microbial functional capacity related to urate handling (Ji et al., 2023). This directly causes insufficient intestinal uricase activity and substantially reduces uric acid degradation efficiency. Undegraded uric acid accumulates in the intestine, increasing the concentration gradient between the intestinal lumen and epithelial cells and promoting diffusion of uric acid into intestinal epithelial cells. It may also affect the function of intestinal uric acid transporters through specific signaling pathways, further aggravating uric acid reabsorption. At the same time, dysbiosis is often accompanied by an increase in harmful bacteria, which may stimulate intestinal epithelial cells by secreting organic acids, amines, and other metabolites, leading to upregulated expression and enhanced activity of uric acid reabsorption transporters such as URAT1 (Sun et al., 2025; Fu et al., 2014). Overgrowth of harmful bacteria can also competitively inhibit the interaction between beneficial bacteria and intestinal epithelial cells, destabilize the intestinal microenvironment, and further drive uric acid reabsorption.

In the dysbiotic state, endotoxins such as LPS released by harmful bacteria can cross the damaged intestinal barrier and enter the bloodstream, inducing local intestinal and systemic inflammatory responses (de Punder and Pruimboom, 2015). Inflammatory mediators such as TNF-α and IL-6 activate signaling pathways including NF-κB, downregulate ABCG2 expression in intestinal epithelial cells, and inhibit uric acid secretion (Herrnreiter et al., 2025). At the same time, inflammation disrupts tight junctions between intestinal epithelial cells, further aggravating barrier dysfunction and forming a vicious cycle of “inflammation-barrier injury-worsened inflammation”, which severely impairs normal uric acid excretion. Ultimately, dysbiosis decreases the production of beneficial metabolites such as SCFAs (Maciel-Fiuza et al., 2023). SCFA deficiency weakens both nutritional support for intestinal epithelial cells and the maintenance of barrier integrity, and it also reduces regulation of uric acid transporters. Together, these effects decrease intestinal uric acid secretion and increase reabsorption.

## 4 Renal injury mediated by gut microbiota-derived metabolites

The major gut microbiota-derived metabolites and microbial pathways potentially involved in HN are summarized in Table 2. Because direct HN-specific human evidence remains limited, the evidence supporting these metabolites should be interpreted according to its source, including CKD studies, HUA/gout studies, animal models, and *in vitro* mechanistic experiments.

**TABLE 2:** Microbial metabolites and microbial pathways potentially involved in hyperuricemic nephropathy.

Microbial metabolite/Pathway	Main microbial source or related process	Proposed effects relevant to HN	Evidence source	Strength of evidence and limitations	Representative references
Short-chain fatty acids (SCFAs; acetate, propionate, butyrate)	Fermentation of dietary fiber by SCFA-producing bacteria	May preserve intestinal barrier integrity, increase tight-junction and mucin expression, reduce gut-derived endotoxemia, regulate inflammatory responses, enhance antioxidant defense through Nrf2-related pathways, and potentially modulate renal or intestinal urate transport	Human CKD studies; animal CKD/HUA/HN-related models; mechanistic and cellular studies	Moderate supportive evidence for gut-kidney protection, but HN-specific human evidence remains limited. Effects may depend on baseline microbiota, diet, and disease stage	Tsuji et al. (2024), Gasaly et al. (2021), Burger-van Paassen et al. (2009), González-Bosch et al. (2021), Li et al. (2022)
Lipopolysaccharide (LPS) and gut-derived endotoxemia	Gram-negative bacteria and increased intestinal permeability during dysbiosis	May activate TLR4/NF-kB signaling in immune and renal cells, promote TNF-alpha, IL-1beta, IL-6, and MCP-1 release, amplify renal inflammation, and aggravate tubular or interstitial injury	Animal and *in vitro* studies; CKD-related evidence; indirect HUA/HN evidence	Biologically plausible and well supported in inflammatory kidney injury, but direct causal evidence in human HN is insufficient	de Punder and Pruimboom (2015), Guijarro-Muñoz et al. (2014), Chowdhury et al. (2006), Su et al. (2021)
Indoxyl sulfate (IS)	Tryptophan-derived indole compounds produced by gut bacteria and converted to IS in the liver	May induce tubular oxidative stress, endoplasmic reticulum stress, mitochondrial dysfunction, apoptosis, inflammatory signaling, and renal interstitial fibrosis	Human and animal CKD studies; renal tubular cell experiments; limited HUA/HN-specific mechanistic evidence	Strong evidence in CKD progression, but mainly extrapolated to HN. Its role in HN should be interpreted as indirect unless validated in HN-specific cohorts or models	Sun et al. (2012), Watanabe et al. (2014), Wang et al. (2004), Qian (2017), Bolati et al. (2013), Cheng et al. (2020), Gelasco and Raymond (2006)
p-Cresyl sulfate (pCS)	Microbial fermentation of tyrosine and phenylalanine, followed by host sulfation	May promote tubular toxicity, oxidative stress, inflammatory activation, impaired tubular repair, and epithelial-mesenchymal transition-related fibrosis	CKD studies; animal and cellular experiments	Evidence is relatively strong in CKD but remains indirect for HN. HN-specific clinical data are scarce	Sun et al. (2012), Watanabe et al. (2014), Qian (2017), Bolati et al. (2011)
Trimethylamine N-oxide (TMAO)	Microbial conversion of dietary choline, phosphatidylcholine, and carnitine to trimethylamine, followed by hepatic oxidation to TMAO	May promote glomerular mesangial activation, TGF-beta/Smad-mediated fibrosis, vascular dysfunction or calcification, and renal hemodynamic injury	CKD, cardiovascular, animal, and mechanistic studies	Relevant to renal injury and fibrosis, but the evidence is largely extrapolated from CKD and cardiovascular disease rather than HN-specific studies	Gupta et al. (2020), Liu et al. (2018), Zhang et al. (2020)
Microbial urate-degrading pathways	Strain-specific bacterial urate degradation pathways and uric acid-metabolizing gene clusters	May contribute to intestinal urate degradation and extra-renal urate elimination, thereby influencing serum uric acid homeostasis	Genomic, mechanistic, and experimental studies; limited human translational evidence	Emerging evidence. Effects are likely strain-specific and should not be generalized to broad bacterial genera without validation	Liu et al. (2023)
Microbial purine metabolism-related pathways	Altered microbial metabolism of amino acids, nucleotides, and purine-related substrates during dysbiosis	May influence purine availability, uric acid production, inflammatory tone, and host metabolic stress, thereby indirectly affecting HUA-related renal injury	Animal HUA models, metabolic inference, and mechanistic studies	Hypothesis-generating evidence. Direct demonstration in human HN is limited; therefore, causal wording should be avoided	Song et al. (2022), Baggott and Tamura (2015), Cader et al. (2020), Wang et al. (2022)
Secondary bile acids and bile acid-related signaling	Conversion of primary bile acids by gut bacteria such as *Bacteroides* and Clostridium-related taxa	May influence intestinal barrier function, microbial ecology, host metabolism, and inflammatory responses relevant to the gut-kidney axis	Gut barrier and metabolism studies; limited kidney disease-specific evidence	Potentially relevant but insufficiently validated in HN. Should be presented as a possible modulatory pathway rather than a proven mechanism	Hofmann and Eckmann (2006), Yu et al. (2021)

Abbreviations: CKD, chronic kidney disease; HN, hyperuricemic nephropathy; HUA, hyperuricemia; IS, indoxyl sulfate; LPS, lipopolysaccharide; MCP-1, monocyte chemoattractant protein-1; Nrf2, nuclear factor erythroid 2-related factor 2; pCS, p-cresyl sulfate; SCFA, short-chain fatty acid; TGF-beta, transforming growth factor-beta; TLR4, Toll-like receptor 4; TMAO, trimethylamine N-oxide.

This table summarizes proposed mechanisms and the corresponding evidence sources. For most metabolites, direct clinical evidence in HN, remains limited; therefore, findings from HUA, gout, CKD, animal models, and *in vitro* studies should be interpreted according to their evidence level.

### 4.1 Reduction of protective metabolites weakens renal defense functions

#### 4.1.1 SCFA deficiency induces gut-derived endotoxemia

Gut microbiota dysbiosis directly impairs microbial metabolic function, one of the most prominent consequences being a marked reduction in the synthesis of protective metabolites such as SCFAs. Multiple studies have shown that fecal SCFA concentrations in patients with CKD (Tsuji et al., 2024) and in animal models (Hall et al., 2020) are significantly lower than those in healthy controls. This change not only disrupts local intestinal metabolic homeostasis but also deprives the host of key “protective signals” provided by the gut microbiota, laying the groundwork for subsequent intestinal barrier disruption, systemic inflammation, and renal injury. As the primary defense preventing intestinal harmful substances from entering the bloodstream, the integrity of the intestinal barrier is essential for systemic health. This barrier consists mainly of a single layer of colonic epithelial cells, intercellular tight junctions (TJs), and the mucus layer on the epithelial surface (Schwarzfischer and Rogler, 2022).

SCFAs are central factors in maintaining intestinal barrier function. First, butyrate is the preferred energy source for colonic epithelial cells; it promotes cellular metabolism and proliferation and maintains epithelial health (Gasaly et al., 2021). Second, butyrate inhibits histone deacetylases (HDACs), thereby upregulating the expression of tight-junction proteins such as occludin, zonula occludens-1 (ZO-1), and claudins, while also regulating their proper localization and strengthening intercellular tightness. In addition, SCFAs stimulate goblet cells to secrete mucins such as MUC2, thicken the mucus layer, and further reinforce the physical barrier (Burger-van Paassen et al., 2009).

When dysbiosis reduces SCFA levels, these protective effects are substantially weakened. Colonic epithelial cells develop dysfunction and even apoptosis due to insufficient energy supply, leading to epithelial damage. Tight-junction protein expression is downregulated and their distribution becomes disordered, loosening intercellular junctions. The mucus layer becomes thinner due to reduced mucin secretion and loses its protective function (Roediger, 1980). Together, these changes impair intestinal barrier function, allowing large amounts of bacterial endotoxins such as LPS, bacterial DNA, and harmful metabolites to readily penetrate the epithelial barrier, enter the portal circulation, spread systemically, and induce “gut-derived endotoxemia” (Lambert, 2008).

#### 4.1.2 Impairment of renal antioxidant capacity and uric acid excretion

As the primary filtering organ, the kidney is among the first organs to encounter these gut-derived toxins. Substances such as LPS reach the kidney via the bloodstream and bind to TLR4 on renal tubular epithelial cells, glomerular mesangial cells, and renal immune cells such as macrophages and dendritic cells. This activates downstream signaling pathways including NF-κB (Chowdhury et al., 2006), initiates transcription of pro-inflammatory genes, and leads to substantial release of pro-inflammatory cytokines such as TNF-α, IL-6, and IL-1β, as well as chemokines such as monocyte chemoattractant protein-1 (MCP-1) (Su et al., 2021). These mediators recruit more inflammatory cells into renal tissue and trigger persistent and excessive local renal inflammation. Chronic inflammation gradually damages glomerular and tubular structures, impairs normal filtration and reabsorption, and significantly weakens renal defense capacity. As a high-oxygen-consuming organ, the kidney generates large amounts of reactive oxygen species (ROS) during filtration and reabsorption. Physiologically, the kidney possesses an integrated antioxidant defense system that removes ROS in a timely manner and maintains cellular redox balance. This system includes antioxidant enzymes such as superoxide dismutase (SOD), glutathione peroxidase (GSH-Px), and catalase (CAT), as well as non-enzymatic antioxidants such as glutathione (GSH), vitamin C, and vitamin E (Kakkar et al., 1997).

SCFAs play an important role in maintaining renal antioxidant capacity. Studies indicate that SCFAs exert antioxidant effects by activating the nuclear factor erythroid 2-related factor 2 (Nrf2) signaling pathway. As one of the most important intracellular antioxidant transcription factors, Nrf2 translocates from the cytoplasm to the nucleus under oxidative stress or specific chemical stimulation, binds to antioxidant response elements (AREs), and initiates downstream expression of antioxidant and detoxifying enzymes (González-Bosch et al., 2021). Butyrate promotes Nrf2 nuclear translocation and activation by inhibiting HDACs or activating GPR43, thereby upregulating the expression and activity of antioxidant enzymes such as SOD and GSH-Px and enhancing renal ROS clearance (Kibbie et al., 2021). When SCFA levels decline, their activation of Nrf2 signaling is weakened, renal endogenous antioxidant enzyme expression and activity decrease substantially, and ROS clearance capacity drops markedly. Meanwhile, the inflammatory response caused by harmful substances such as intestinal LPS further stimulates renal cells to generate large amounts of ROS (Yang et al., 2007).

When ROS accumulation exceeds renal clearance capacity, oxidative stress occurs. Excess ROS are highly chemically reactive and attack biological macromolecules such as lipids, proteins, and DNA in renal cells. They disrupt the integrity and fluidity of cell membranes, causing cellular leakage or even death; alter protein structure, leading to loss of function or gain of toxicity and impairing enzymatic and signaling functions; and cause DNA strand breaks and base modifications, inducing mutations or apoptosis (Nowzari et al., 2000). Such injuries broadly affect glomeruli, tubules, and the renal interstitium, causing glomerulosclerosis, tubular atrophy, and interstitial fibrosis, and severely impairing core renal protective functions such as filtration, reabsorption, and endocrine regulation.

Uric acid is the final product of human purine metabolism, and its excretion depends mainly on the kidney. Renal uric acid excretion is a complex process involving glomerular filtration, tubular reabsorption, secretion, and post-secretory reabsorption, among which tubular transport is decisive. This process is mediated by a series of specific transporters: URAT1, encoded by SLC22A12, and glucose transporter 9 (GLUT9), which mediate uric acid reabsorption; and ABCG2, encoded by ABCG2, along with organic anion transporters 1/3 (OAT1/3), which mediate uric acid secretion (Chung and Kim, 2021). Under physiological conditions, these transporters act synergistically to maintain the balance of uric acid excretion. SCFAs can regulate the expression and activity of renal uric acid transporters through specific receptors such as GPR43 and GPR41. Studies show that SCFAs may inhibit URAT1 function and reduce tubular uric acid reabsorption, while promoting the expression and membrane localization of ABCG2 and OAT1/3, thereby enhancing tubular secretion. Through this bidirectional regulation, SCFAs promote renal uric acid excretion and maintain stable serum uric acid levels (Li et al., 2022).

When dysbiosis reduces SCFA levels, this normal regulation of uric acid transporters weakens or disappears. URAT1-mediated uric acid reabsorption increases relatively, whereas ABCG2-and OAT1/3-mediated uric acid secretion decreases markedly (Li et al., 2022). Net tubular uric acid reabsorption therefore increases, ultimately impairing uric acid excretion, elevating serum uric acid, and inducing hyperuricemia. Hyperuricemia is not only the major cause of gout but also an important risk factor for renal injury. When serum uric acid exceeds its solubility threshold, urate crystals precipitate and deposit in renal tubules, the interstitium, and the renal pelvis, directly obstructing tubules and impairing urine formation and excretion. These crystals can also act as “danger signals” that activate renal inflammatory cells and resident renal cells (Mei et al., 2022).

Urate crystals can activate pathways such as the NLRP3 inflammasome, induce the production and release of pro-inflammatory cytokines such as IL-1β, and trigger strong inflammatory responses. Uric acid itself also has the capacity to induce oxidative stress and promote ROS generation (Kim et al., 2020). Urate crystal deposition, inflammation, and oxidative stress mutually reinforce one another, forming a vicious cycle that leads to tubular injury, interstitial inflammation, and fibrosis, further damaging renal structure and function and accelerating renal functional decline (Khan, 2004).

### 4.2 Increased harmful metabolites aggravate renal pathological injury

#### 4.2.1 Direct injury of renal tubules and glomeruli by harmful metabolites

Through metabolism of host-derived nutritional precursors or through its own metabolic disturbance, dysbiotic gut microbiota produces multiple harmful metabolites, among which trimethylamine N-oxide (TMAO), indoxyl sulfate (IS), and p-cresyl sulfate (pCS) are the most representative. These harmful substances systematically aggravate renal pathology through multiple interrelated and progressively amplified pathophysiological pathways. After active uptake or passive absorption by renal cells, they accumulate intracellularly and exert direct toxic effects, disrupting normal renal structure and function (Sun et al., 2012).

Renal tubular epithelial cells (TECs) are the main executors of renal reabsorption and secretion and are also major targets of injurious metabolites. IS and pCS can be actively taken up by TECs through organic anion transporters (OATs), especially OAT1 and OAT3 (Watanabe et al., 2014).

After entering cells, high concentrations of IS and pCS exert toxicity by inducing massive ROS generation and oxidative stress. They also activate endoplasmic reticulum stress and mitochondrial apoptotic pathways, thereby inducing apoptosis of renal tubular epithelial cells.

IS can trigger apoptosis by activating the c-Jun N-terminal kinase (JNK) and p38 mitogen-activated protein kinase (p38 MAPK) signaling pathways, promoting the expression of pro-apoptotic proteins such as Bax and suppressing anti-apoptotic proteins such as Bcl-2 (Wang et al., 2004). These metabolites can also inhibit the proliferation and migration of tubular epithelial cells, impair post-injury repair, and ultimately cause tubular atrophy and functional loss.

The glomerulus is the core filtration structure of the kidney and consists of mesangial cells, podocytes, and endothelial cells. Injurious metabolites such as TMAO and IS can also damage glomerular cells. TMAO promotes glomerular mesangial cell proliferation and excessive mesangial matrix deposition. Studies show that TMAO treatment upregulates pro-proliferative and pro-fibrotic cytokines such as TGF-β and platelet-derived growth factor (PDGF) in mesangial cells, accelerating glomerulosclerosis (Gupta et al., 2020). IS directly injures podocytes, which are highly specialized cells whose foot processes form a key component of the filtration barrier. IS induces ROS generation and activates inflammatory signaling in podocytes, causing foot-process effacement and apoptosis, disrupting filtration barrier integrity, and resulting in proteinuria (Jia et al., 2024).

#### 4.2.2 Cascade amplification of inflammation, oxidative stress, and fibrosis

Inflammation is a core driver of renal injury progression. Elevated levels of injurious metabolites play a key role in inducing and aggravating systemic chronic low-grade inflammation and local renal inflammatory responses (Qian, 2017).

As endogenous damage-associated molecular patterns (DAMPs), IS and pCS activate immune cells by binding to pattern-recognition receptors (PRRs) on their surface. IS specifically binds TLR4, a major PRR widely expressed on macrophages, monocytes, dendritic cells, and resident renal cells. The IS-TLR4 interaction activates downstream pathways such as NF-κB and MAPK, leading to substantial release of pro-inflammatory cytokines (TNF-α, IL-6, IL-1β) and chemokines (MCP-1) (Su et al., 2021). These mediators recruit circulating monocytes, macrophages, and other inflammatory cells into renal tissue, initiating and amplifying local inflammation. LPS further activates systemic and renal immune cells and aggravates inflammation, playing a key role in CKD progression. Oxidative stress and renal interstitial fibrosis are two core pathological features of CKD progression toward ESRD, and injurious metabolites significantly promote the entire process.

Oxidative stress is cellular injury caused by an imbalance between ROS generation and clearance. IS and pCS are potent inducers of oxidative stress and can stimulate ROS production in renal cells through multiple pathways. IS acts in part by inhibiting the Nrf2-ARE signaling pathway. Nrf2 is the most critical intracellular antioxidant transcription factor; under oxidative stress, it translocates from the cytoplasm to the nucleus, binds AREs, and initiates the transcription of antioxidant and detoxifying enzymes such as SOD, GSH-Px, and CAT (Bolati et al., 2013). IS suppresses Nrf2 nuclear translocation and activity, reduces antioxidant enzyme expression and activity, and diminishes ROS clearance. At the same time, inflammation induced by IS and pCS further stimulates ROS production.

Excess ROS not only directly damage lipids, proteins, and DNA in renal cells but also activate multiple pro-fibrotic and pro-inflammatory signaling pathways, further aggravating renal injury (Bolati et al., 2013; Andrews et al., 2025).

Renal interstitial fibrosis is a common pathological outcome of CKD and is characterized by activation and proliferation of interstitial fibroblasts and excessive deposition of extracellular matrix (ECM) components such as collagen. TMAO, IS, and pCS all significantly promote renal interstitial fibrosis (Liu et al., 2018). TMAO acts by activating the TGF-β/Smad signaling pathway, and TGF-β is one of the most potent pro-fibrotic cytokines.

TMAO treatment upregulates TGF-β expression in renal tissue. After TGF-β binds to its receptor, it activates Smad2 and Smad3; phosphorylated Smad2/3 forms a complex with Smad4, translocates into the nucleus, and may regulate the expression of pro-fibrotic genes such as α-smooth muscle actin (α-SMA) and type I/III collagen (Sun et al., 2012). IS and pCS can also stimulate epithelial-mesenchymal transition (EMT) in renal tubular epithelial cells (Bolati et al., 2011). During EMT, tubular epithelial cells lose epithelial characteristics, such as decreased E-cadherin expression, and acquire mesenchymal features, such as increased α-SMA and vimentin expression. The transdifferentiated cells display fibroblast-like functions and secrete large amounts of ECM. In addition, these metabolites directly activate renal interstitial fibroblasts, promote their proliferation and collagen synthesis, and may ultimately contribute to renal interstitial fibrosis, disrupting normal renal structure and function.

#### 4.2.3 Hemodynamic disturbance and vascular calcification exacerbate renal injury

Injurious metabolites may also indirectly aggravate renal injury by affecting renal hemodynamics and vascular structure. IS can alter glomerular hemodynamics. Studies show that IS constricts both afferent and efferent arterioles, with a more pronounced effect on efferent arterioles (Ichii et al., 2014). This constriction increases intraglomerular pressure and temporarily increases the glomerular filtration rate (GFR). Over the long term, sustained intraglomerular hypertension damages glomerular capillary endothelial cells and the basement membrane, disrupts the filtration barrier, causes glomerulosclerosis, and eventually leads to irreversible GFR decline.

The role of TMAO in vascular calcification has been reported primarily in CKD and cardiovascular contexts. Vascular calcification contributes to the high cardiovascular burden in CKD and may also affect renal perfusion. TMAO has been shown to activate the bone morphogenetic protein 2 (BMP2)-Smad signaling pathway, promote differentiation of vascular smooth muscle cells (VSMCs) into osteoblast-like cells, and upregulate osteogenic transcription factors such as Runx2 (Zhang et al., 2020). This causes VSMCs to express osteogenic markers such as alkaline phosphatase and osteopontin while suppressing smooth-muscle markers such as alpha-SMA. Calcification of renal vessels may reduce renal perfusion and aggravate renal ischemic injury. These mechanisms are summarized in the integrated mechanism diagram in Figure 2.

To avoid redundancy between separate diagrams, the urate-metabolism pathway and the metabolite/barrier-mediated renal injury pathway are combined into Figure 2. This integrated design clarifies that these mechanisms are parallel but interacting routes that converge on renal inflammation, oxidative stress, tubular injury, and fibrosis.

## 5 Roles of intestinal barrier dysfunction and gut-kidney axis interactions in the pathogenesis of hyperuricemic nephropathy

### 5.1 Imbalance in bidirectional regulation between the gut microbiota and the intestinal barrier

#### 5.1.1 Maintenance of the intestinal barrier by healthy microbiota

The gut microbiota and intestinal barrier interact closely in a bidirectional manner, forming a dynamic regulatory network. On the one hand, a healthy gut microbiota actively maintains intestinal barrier function through multiple pathways. SCFAs, especially butyrate, produced by beneficial bacteria are the main energy source for intestinal epithelial cells, promoting epithelial proliferation and differentiation and maintaining epithelial integrity (Gasaly et al., 2021). SCFAs also inhibit HDACs, upregulate the expression of tight-junction proteins such as occludin, ZO-1, and claudins, and strengthen tight-junction integrity (Tsuji et al., 2024). In addition, beneficial bacteria stimulate goblet cells to secrete mucins, thicken the mucus layer, and reinforce the physical barrier. They can also activate host-cell signaling pathways such as aryl hydrocarbon receptor (AHR) and Nrf2, promoting the secretion of antimicrobial peptides such as defensins and cryptdins by intestinal epithelial cells and Paneth cells and enhancing the bactericidal capacity of the chemical barrier (Palrasu et al., 2025).

#### 5.1.2 Disruption of the intestinal barrier by dysbiosis

Intestinal barrier integrity also provides essential survival conditions for the gut microbiota. The mucus layer on the surface of intestinal epithelial cells and the intercellular spaces provide sites for microbial colonization and growth. An intact intestinal barrier prevents gut microorganisms and their metabolites from entering the bloodstream, thereby avoiding systemic inflammation (Pastorelli et al., 2013). Substances secreted by intestinal epithelial cells, including antimicrobial peptides and mucus, selectively inhibit the growth of certain bacteria and regulate microbial composition and abundance. During gut microbiota dysbiosis, this bidirectional balance is disrupted. The protective effect of the microbiota on the intestinal barrier weakens, while harmful bacteria and their metabolites directly or indirectly attack the layers of the intestinal barrier, ultimately causing barrier dysfunction (Yu, 2018). Dysbiosis compromises intestinal barrier integrity and function through multiple interrelated and mutually reinforcing mechanisms at the physical, chemical, and immune levels.

### 5.2 Multilevel injury mechanisms of intestinal barrier dysfunction

#### 5.2.1 Physical barrier injury

The physical barrier is the outermost defensive line of the intestinal barrier. It consists of a single layer of columnar intestinal epithelial cells, intercellular tight junctions, and the epithelial surface mucus layer, and it is the major obstacle preventing harmful substances from entering the body. Intestinal epithelial cells are the core component of the physical barrier, and their integrity and viability directly determine barrier function. During dysbiosis, pathogenic bacteria such as enteropathogenic and enterohemorrhagic *Escherichia coli* (EPEC and EHEC) may overproliferate, adhere to epithelial receptors through surface adhesins, and colonize the intestine (Isaacson, 1983). They then secrete various toxins that directly damage epithelial cells. The inflammatory response induced by dysbiosis produces large amounts of pro-inflammatory cytokines, including TNF-α, IL-1β, and IL-6, which can induce epithelial-cell apoptosis by activating intracellular apoptotic pathways such as Fas/FasL and TNF/TNFR and simultaneously inhibit epithelial proliferation and repair, further aggravating epithelial damage (Su et al., 2021). In dysbiosis, pathogenic bacteria and their metabolites, such as LPS, can bind TLR4 on intestinal epithelial cells and immune cells, activate NF-κB signaling, and induce abundant release of inflammatory mediators such as TNF-α and IL-1β (Gasaly et al., 2021). These mediators disrupt tight junctions through several mechanisms: downregulating the mRNA and protein expression of tight-junction proteins such as occludin, ZO-1, and claudins; inducing phosphorylation of tight-junction proteins; altering their membrane localization from the cell membrane to the cytoplasm, thereby loosening junctions; and activating matrix metalloproteinases (MMPs) to degrade intercellular junctional structures (Burger-van Paassen et al., 2009).

Metabolic imbalance and inflammation caused by dysbiosis increase intestinal ROS generation (Bolati et al., 2013). Excess ROS oxidatively modify tight-junction proteins and impair their structure and function. ROS also activate MAPK signaling pathways, further downregulate tight-junction protein expression, and aggravate junctional disruption (Ha et al., 2007).

The mucus layer is a gel-like substance covering intestinal epithelial cells and consists mainly of mucins, especially MUC2, secreted by goblet cells. It can be divided into an inner sterile layer closely attached to epithelial cells and an outer layer containing microbiota (Johansson et al., 2011). It lubricates the intestine and prevents microorganisms and harmful substances from directly contacting epithelial cells, serving as an important supplement to the physical barrier (Grondin et al., 2020). During dysbiosis, decreased beneficial bacteria and increased pathogenic bacteria inhibit goblet-cell proliferation and differentiation. Pathogens such as *Pseudomonas aeruginosa* in the Proteobacteria phylum may secrete mucinases, including proteases and glycosidases, which degrade the protein backbone or glycan chains of mucins, disrupt mucus-layer integrity, and cause the mucus layer to become thin and damaged, thereby losing its protective effect on epithelial cells (Deplancke and Gaskins, 2001).

#### 5.2.2 Chemical barrier injury

The chemical barrier consists of multiple antimicrobial substances secreted by intestinal epithelial cells, Paneth cells, goblet cells, and related cells. These substances include antimicrobial peptides such as defensins, cryptdins, and regenerating islet-derived proteins (RegIII), digestive enzymes such as lysozyme and proteases, mucins, and bile acids. They maintain intestinal microecological balance by directly killing bacteria and inhibiting the adhesion and colonization of pathogens. Dysbiosis can impair chemical barrier function through multiple pathways. Antimicrobial peptides are among the most critical antibacterial components of the chemical barrier, and their expression and secretion are precisely regulated by the gut microbiota (Jin et al., 2025). Beneficial bacteria such as *Lactobacillus* and Bifidobacterium promote antimicrobial peptide production through multiple signaling pathways. When beneficial bacteria decrease during dysbiosis, activation of these pathways weakens, antimicrobial peptide expression and secretion decrease significantly, and the bactericidal capacity of the chemical barrier is impaired (Huang et al., 2017). Bile acids, important chemical barrier substances synthesized by the liver, inhibit the growth of some intestinal pathogens by disrupting bacterial cell membrane integrity (Hofmann and Eckmann, 2006). The gut microbiota plays a key role in bile acid metabolism by converting primary bile acids into secondary bile acids with stronger antimicrobial activity. During dysbiosis, the abundance of bacteria involved in bile acid conversion, such as *Clostridium* and *Bacteroides* species, declines, reducing secondary bile acid production and weakening antimicrobial effects (Yu et al., 2021). At the same time, some pathogens such as *Enterococcus* can produce bile salt hydrolases that degrade and inactivate bile acids. Lysozyme, an enzyme that hydrolyzes bacterial cell walls, is mainly secreted by Paneth cells and intestinal epithelial cells and has broad-spectrum antibacterial activity. Dysbiosis can suppress Paneth-cell function, reduce lysozyme secretion, and lower the bactericidal ability of the chemical barrier (Kim JE. et al., 2022).

#### 5.2.3 Immune barrier injury

The immune barrier is an important component of the intestinal barrier and consists of gut-associated lymphoid tissue (GALT), immune cells, and cytokines and antibodies secreted by these cells. Its core functions are to recognize and eliminate pathogens while maintaining immune tolerance toward food antigens and commensal bacteria. During dysbiosis, pathogens and their metabolites, including LPS and peptidoglycan, can bind PRRs such as TLR4, TLR2, and NOD-like receptors (NLRs) on innate immune cells such as dendritic cells and macrophages, activating these cells and inducing the release of large amounts of pro-inflammatory cytokines (TNF-α, IL-1β, IL-6, IL-12) and chemokines (MCP-1, IL-8) (Su et al., 2021). Excessive inflammation directly damages intestinal epithelial cells and tight junctions, disrupts immune tolerance, and induces abnormal immune responses against commensal bacteria and food antigens. Dysbiosis may also alter dendritic-cell maturation and antigen presentation, thereby changing T-cell differentiation (Bashir et al., 2023). The intestinal barrier is the first line of defense in the gut-kidney axis. It consists of the intestinal epithelial cell layer, tight junctions, and mucus layer, and its core function is to selectively allow nutrients and water to pass while preventing bacteria, endotoxins, and antigens from entering the bloodstream. Dysbiosis substantially changes the metabolic profile and generates excessive uremic toxins. These toxins are difficult for the kidney to clear completely, accumulate in the body, increase the renal metabolic burden, and induce or aggravate renal injury.

### 5.3 Renal injury cascades mediated by gut-kidney axis dysregulation

#### 5.3.1 Direct renal injury caused by uremic toxin accumulation

Some gut bacteria, including certain Proteobacteria, can decompose dietary tryptophan through tryptophanase to produce indole and indole-3-acetic acid. These indole compounds enter the liver via the portal vein and are converted into indoxyl sulfate (IS) by sulfotransferases. Accumulated IS can injure the kidney through multiple pathways: inducing oxidative stress and endoplasmic reticulum stress in renal tubular epithelial cells and promoting apoptosis; activating renal interstitial fibroblasts, stimulating synthesis of collagen and other ECM components, and accelerating renal interstitial fibrosis; and activating TLR4/NF-κB signaling, thereby promoting renal inflammation (Cheng et al., 2020). Gut microbiota dysbiosis can activate systemic and local renal immune-inflammatory responses through multiple pathways. These inflammatory responses not only directly injure renal tissue but also further disrupt the intestinal barrier, forming a vicious cycle of “intestinal injury-renal injury”.

#### 5.3.2 Immune-inflammatory activation and circulating immune injury

After intestinal barrier injury, microbial antigens and endotoxins in the intestine activate dendritic cells and macrophages in the intestinal mucosa. These activated antigen-presenting cells migrate to the kidney via the lymphatic and blood circulation, where they present antigens locally and activate renal T cells and B cells (Bashir et al., 2023). Activated T cells differentiate into Th1 and Th17 effector cells and secrete large amounts of pro-inflammatory mediators such as IFN-γ and IL-17, directly damaging renal cells (Summers et al., 2009). Meanwhile, activated T cells can promote antibody production. These antibodies may bind renal tissue antigens to form immune complexes, which deposit in glomeruli or the renal interstitium and induce immune-complex glomerulonephritis or interstitial nephritis.

Gut-derived inflammatory signals, including endotoxins and microbial antigens, can stimulate resident renal cells to secrete large amounts of pro-inflammatory cytokines (TNF-α, IL-6, IL-1β) and chemokines (MCP-1, CXCL8) (Su et al., 2021). These mediators further recruit neutrophils, monocytes, lymphocytes, and other inflammatory cells into renal tissue, forming an amplified inflammatory milieu.

Although gut microbiota dysbiosis has been implicated in immune dysregulation in several autoimmune kidney diseases, its direct relevance to HN remains unclear. Therefore, autoimmune mechanisms should currently be regarded as speculative and not as established pathways in HN (Zhang L. et al., 2025). The discussion of antineutrophil cytoplasmic antibody (ANCA)-associated vasculitis is therefore retained only as an example of microbiota-immune interactions in kidney disease, not as evidence for a validated HN mechanism (Yu et al., 2022).

#### 5.3.3 Water-salt metabolic disorders and oxidative stress aggravate renal injury

The gut microbiota plays a key role in regulating intestinal water and salt absorption by producing SCFAs that influence epithelial ion transport and promote sodium excretion. During dysbiosis, this regulatory function is impaired, which may increase intestinal sodium absorption and induce water and sodium retention (Montrose and Chu, 1997). Dysbiosis can also activate the renin-angiotensin-aldosterone system (RAAS), further aggravating water and sodium retention. RAAS activation increases renal reabsorption of sodium and water, raises blood volume and blood pressure, and increases renal hemodynamic burden (Lu et al., 2020). Long-term hypertension and hypervolemia induce glomerular hyperfiltration and hyperperfusion, accelerating glomerulosclerosis and renal functional decline. In addition, dysbiosis may disturb intestinal potassium absorption and excretion, causing hyperkalemia or hypokalemia, and electrolyte imbalance can also impair renal function (Ondrussek-Sekac et al., 2021). Because these water-salt and RAAS-related mechanisms are not well validated in HN-specific cohorts, they should be interpreted as auxiliary or extrapolated pathways rather than core HN mechanisms.

Oxidative stress is a key mechanism of renal injury, and dysbiosis aggravates renal oxidative stress through multiple pathways. First, endotoxemia induces renal cells to generate large amounts of ROS, which directly damage renal cellular DNA, proteins, and lipids, leading to cellular dysfunction and death (Poston and Koyner, 2019). Second, uremic toxins such as IS, pCS, and TMAO also induce ROS generation in renal cells and aggravate oxidative stress; IS promotes ROS production by activating NADPH oxidase (Gelasco and Raymond, 2006). In addition, dysbiosis reduces SCFAs and weakens their antioxidant effects. SCFAs reduce ROS accumulation by upregulating antioxidant enzymes such as SOD and CAT (Sun et al., 2022) and SCFA deficiency further intensifies renal oxidative stress. Long-term oxidative stress induces chronic renal tissue injury, including glomerulosclerosis and tubulointerstitial fibrosis, and ultimately leads to renal failure. These reciprocal processes and their potential intervention points are summarized in Figure 3.

## 6 Research perspectives and challenges

### 6.1 Establishing causality beyond association

Although increasing evidence suggests a close relationship between gut microbiota dysbiosis and HN, the causal role of the gut microbiota in the initiation and progression of HN remains insufficiently defined. Most available human studies (Zho et al., 2024; Shao et al., 2017; Song et al., 2022; Ji et al., 2023; Miyajima et al., 2024) are cross-sectional and can only demonstrate associations between microbial composition, serum uric acid levels, and renal injury markers (Shao et al., 2017; Ji et al., 2023; Miyajima et al., 2024). Such studies are vulnerable to confounding by diet, age, sex, obesity, medication use, comorbid metabolic diseases, baseline kidney function, and geographic or ethnic background (Miyajima et al., 2024; Healey et al., 2017). Therefore, microbial alterations observed in HUA, gout, CKD, or HN should not be interpreted as direct pathogenic mechanisms without appropriate validation (Zho et al., 2024; Shao et al., 2017; Song et al., 2022; Tsuji et al., 2024).

Future studies should place greater emphasis on distinguishing correlation from causation. Longitudinal human cohorts (Zho et al., 2024; Miyajima et al., 2024) are needed to determine whether specific microbial signatures precede the onset of HUA and HN or merely reflect established renal dysfunction (Miyajima et al., 2024; Zhao et al., 2024). Mechanistic studies using germ-free animals, antibiotic-treated models (Song et al., 2022), gnotobiotic colonization, fecal microbiota transplantation (Miyajima et al., 2024), and strain-specific intervention models may help identify whether particular microbial communities or functional genes directly influence urate metabolism, intestinal urate excretion, inflammation, and renal fibrosis (Zho et al., 2024; Song et al., 2022; Khan, 2004; Yuan et al., 2025). Integration of human data with animal experiments and *in vitro* validation will be essential for constructing a more rigorous causal framework (Zho et al., 2024; Song et al., 2022; Miyajima et al., 2024).

### 6.2 Bidirectional gut-kidney interactions in HN progression

A major challenge in this field is that the gut-kidney axis should be considered a bidirectional regulatory network rather than a unidirectional pathway from gut dysbiosis to renal injury (Chen et al., 2022; Liu et al., 2024; Cheng et al., 2020). In the early stage of HN, dysbiosis may contribute to hyperuricemia, intestinal barrier disruption, endotoxemia, accumulation of microbial metabolites, and renal inflammatory injury (Zho et al., 2024; Song et al., 2022; Tsuji et al., 2024; Li et al., 2022). However, once renal dysfunction develops, impaired renal clearance can lead to the retention of urea, uric acid, indoxyl sulfate, p-cresyl sulfate, TMAO, and other metabolites, which may further alter the intestinal microenvironment and aggravate dysbiosis (Tsuji et al., 2024; Sun et al., 2012; Qian, 2017; Andrews et al., 2025; Cheng et al., 2020).

Renal dysfunction may also promote gut barrier injury through several indirect mechanisms, including uremia-related intestinal epithelial stress, altered intestinal pH, increased intestinal urea diffusion and ammonia generation, systemic inflammation, oxidative stress, dietary restriction, reduced physical activity, and exposure to medications such as urate-lowering agents, antibiotics, diuretics, phosphate binders, or immunomodulatory drugs (Tsuji et al., 2024; Andrews et al., 2025; Cheng et al., 2020; Ondrussek-Sekac et al., 2021). These factors can reshape microbial composition and function, thereby forming a vicious cycle of renal injury, toxin retention, intestinal barrier disruption, and further dysbiosis (Chen et al., 2022; Tsuji et al., 2024; Cheng et al., 2020). Future research should therefore evaluate both directions of the gut-kidney axis and determine whether interrupting this feedback loop can slow HN progression (Zho et al., 2024; Tsuji et al., 2024; Cheng et al., 2020).

### 6.3 Microbiota-targeted interventions and translational limitations

The gut microbiota represents a promising therapeutic target, but microbiota-based strategies for HN remain at an exploratory stage (Liu P. et al., 2025; Yuan et al., 2025; Othman et al., 2025). Probiotics and synbiotics may influence urate homeostasis by promoting urate degradation, increasing SCFA production, improving intestinal barrier integrity, and modulating inflammatory responses (Sun et al., 2025; Liu P. et al., 2025; Othman et al., 2025). However, the effects of probiotics are likely to be strain-specific, dose-dependent, and influenced by baseline microbiota composition (Zhao et al., 2024; Othman et al., 2025; Healey et al., 2017). At present, there is insufficient clinical evidence to recommend any specific probiotic strain as a standard therapy for HN (Liu P. et al., 2025; Yuan et al., 2025; Othman et al., 2025).

Prebiotics, dietary fiber, resistant starch, and dietary patterns enriched in plant-based foods may improve microbial fermentation and increase SCFA production, thereby supporting barrier integrity and reducing inflammation (González-Bosch et al., 2021; Li et al., 2022; Sun et al., 2022; Healey et al., 2017). Dietary interventions, including reduced excessive purine intake, limitation of fructose and alcohol, adequate fiber intake, and weight management, may indirectly modulate both urate metabolism and the gut microbiota (Sun et al., 2025; Li et al., 2022; Healey et al., 2017). Nevertheless, it remains difficult to separate microbiota-mediated effects from broader metabolic benefits (Healey et al., 2017). Future clinical trials should include microbiome, metabolomic, renal, and urate-related endpoints to clarify the contribution of gut microbiota modulation (Miyajima et al., 2024; Zhao et al., 2024; Healey et al., 2017).

Fecal microbiota transplantation provides an important experimental tool for testing causality, but its clinical application in HN is not yet established (Zho et al., 2024; Yuan et al., 2025). Safety concerns, donor selection, long-term ecological stability, risk of pathogen transmission, and interindividual response variability limit its current clinical use (Yuan et al., 2025; Healey et al., 2017). Postbiotic and metabolite-targeting approaches, such as SCFA supplementation or strategies to reduce indoxyl sulfate, p-cresyl sulfate, TMAO, and endotoxin burden, may represent more controllable therapeutic directions (Tsuji et al., 2024; González-Bosch et al., 2021; Sun et al., 2012; Qian, 2017; Liu et al., 2018). However, these approaches require rigorous evaluation in HN-specific models and well-designed clinical trials before translation into routine practice (Liu P. et al., 2025; Yuan et al., 2025; Othman et al., 2025).

Figure 3 summarizes the bidirectional feedback loop and highlights intervention points that correspond to the therapeutic strategies discussed above. Importantly, the figure distinguishes plausible or experimental approaches from strategies that still require HN-specific clinical validation.

### 6.4 Predictive biomarkers and precision stratification

Another important research direction is the identification of microbiota-related biomarkers for risk prediction, disease stratification, and therapeutic monitoring (Miyajima et al., 2024; Zhao et al., 2024). Potential biomarkers include microbial taxa, urate-degrading functional genes, genes involved in purine metabolism, intestinal barrier-associated markers, fecal and circulating SCFAs, LPS, indoxyl sulfate, p-cresyl sulfate, TMAO, bile acid profiles, and inflammatory mediators (Sun et al., 2025; Liu et al., 2023; Tsuji et al., 2024; González-Bosch et al., 2021; Qian, 2017; Bashir et al., 2023). These markers should be evaluated together with conventional clinical indicators such as serum uric acid, urinary uric acid excretion, eGFR, proteinuria, serum creatinine, cystatin C, and renal imaging or pathological findings when available (Miyajima et al., 2024).

Multi-omics approaches combining metagenomics, metatranscriptomics, metabolomics, proteomics, and host genomic information may help identify microbial signatures that are more functionally meaningful than taxonomic changes alone (Sun et al., 2025; Miyajima et al., 2024; Zhao et al., 2024). Machine-learning models may improve prediction of HUA-to-HN transition or HN progression, but such models must be externally validated in independent, multicenter cohorts (Miyajima et al., 2024; Zhao et al., 2024). Without validation, microbiome-based prediction may suffer from overfitting and limited generalizability (Miyajima et al., 2024; Zhao et al., 2024; Healey et al., 2017).

### 6.5 Interindividual variability and personalized intervention

Substantial interindividual variability is a major obstacle to the clinical translation of microbiota-based strategies (Zhao et al., 2024; Healey et al., 2017). Baseline microbial composition, habitual diet, ethnicity, age, sex, body mass index, renal function stage, host genetic background, urate transporter polymorphisms, medication exposure, and comorbidities such as diabetes, hypertension, obesity, and gout may all influence microbiota composition and treatment response (Leask et al., 2020; Asma Sakalli et al., 2023; Zhang Y. et al., 2025; Chung and Kim, 2021; Zhao et al., 2024; Healey et al., 2017). Therefore, a uniform microbiota-targeted intervention is unlikely to be effective for all patients (Zhao et al., 2024; Othman et al., 2025; Healey et al., 2017).

Future studies should develop personalized intervention strategies based on microbial enterotypes, metabolic phenotypes, renal function status, and urate-handling characteristics (Miyajima et al., 2024; Zhao et al., 2024; Healey et al., 2017). Adaptive trial designs may be useful for identifying responder subgroups and for determining whether baseline microbial signatures can predict responses to probiotics, prebiotics, dietary modification, urate-lowering therapy, or metabolite-targeting interventions. Such precision approaches may ultimately improve the feasibility and effectiveness of microbiota-centered management in HN (Miyajima et al., 2024; Zhao et al., 2024).

### 6.6 Future study design and clinical endpoints

To improve the quality of evidence, future studies should adopt standardized diagnostic criteria for HN and clearly distinguish HN from asymptomatic hyperuricemia, gout, uric acid nephrolithiasis, and general CKD (Stack et al., 2019; Mei et al., 2022; Miyajima et al., 2024). Study designs should also carefully record diet, medication use, antibiotic exposure, renal function, urate-lowering therapy, and comorbid diseases (Miyajima et al., 2024; Zhao et al., 2024; Healey et al., 2017). In addition to microbiome composition, functional and metabolic readouts should be incorporated to determine whether microbial changes are biologically relevant (Sun et al., 2025; Tsuji et al., 2024; Sun et al., 2012; Qian, 2017; Miyajima et al., 2024).

Clinical trials should include clinically meaningful renal outcomes, such as change in eGFR, albuminuria or proteinuria, serum creatinine, serum uric acid, urinary uric acid excretion, inflammatory biomarkers, and, where feasible, renal histological or imaging endpoints (Stack et al., 2019; Mei et al., 2022; Khan, 2004; Miyajima et al., 2024). Long-term follow-up is needed to determine whether microbiota-targeted interventions can modify disease progression rather than only transiently alter microbial composition (Liu P. et al., 2025; Yuan et al., 2025; Healey et al., 2017). Overall, future research should move from descriptive association studies toward mechanism-guided, HN-specific, longitudinal and interventional studies (Zho et al., 2024; Miyajima et al., 2024; Yuan et al., 2025). Such efforts will be essential for translating gut microbiota research into reliable biomarkers and clinically applicable therapeutic strategies for HN (Yuan et al., 2025; Othman et al., 2025; Healey et al., 2017).
